# Injection site reaction to teclistamab in a patient with multiple myeloma

**DOI:** 10.1016/j.jdcr.2024.10.013

**Published:** 2024-11-02

**Authors:** Isabel C. Yoon, Ngan Do, Thomas Vazquez, David E. Elder, Katherine T. Steele, Misha Rosenbach

**Affiliations:** a Perelman School of Medicine, University of Pennsylvania, Philadelphia, Pennsylvania; b Department of Dermatology, University of Pennsylvania, Philadelphia, Pennsylvania; c Department of Pathology and Laboratory Medicine, Hospital of the University of Pennsylvania, Philadelphia, Pennsylvania

**Keywords:** bispecific antibodies, bispecific T-cell engagers, drug reaction, injection site reaction, multiple myeloma, teclistamab

## Introduction

Bispecific antibodies are emerging as promising novel treatments for malignancies. As a subclass of bispecific antibodies, bispecific T-cell engaging antibodies (BiTE) target a tumor antigen while also activating T cells via CD3 engagement.

Teclistamab (Tecvayli, Janssen Biotech, Inc) is a BiTE with Food and Drug Administration approval for relapsed or refractory multiple myeloma. With targets for CD3 on T-cells and B-cell maturation antigen (BCMA) on myeloma cells, teclistamab activates T cells while also inducing lysis of myeloma cells.^1^ The most common adverse events of teclistamab reported in MajesTEC-1, a phase 1-2 study, were infections, cytopenias, and cytokine release syndrome (CRS). Dermatologic adverse events (DAEs) were reported in 36.4% of patients and characterized as an injection site reaction of grade I or II severity (Common Terminology Criteria for Adverse Events).^1^

Follow-up studies of teclistamab have not reported injection site reactions or other DAEs.^2^, ^3^, ^4^, ^5^ Studies evaluating teclistamab in combination with daratumumab (TRIMM-2) or daratumumab and lenalidomide (MajesTEC-2) have also not reported DAEs.^6^^,^^7^ Moreover, to date, there have been no studies characterizing the specific dermatologic and histopathologic features of an injection site reaction to teclistamab.

In this report, we describe a patient who developed an injection site reaction following teclistamab administration.

## Case report

A 66-year-old with a history of multiple myeloma was admitted for teclistamab initiation. She was first diagnosed with IgM Kappa marginal zone lymphoma with plasmacytic differentiation. Despite receiving several different lines of treatment, the patient experienced disease progression and eventually met criteria for multiple myeloma. The decision was made to start teclistamab with planned step-up dosing. During her admission, the patient received dexamethasone premedication and allopurinol in addition to teclistamab. Two days following her first dose of teclistamab, she was found to have a new lesion on her abdomen at the site of her teclistamab injection.

**Fig 1:**
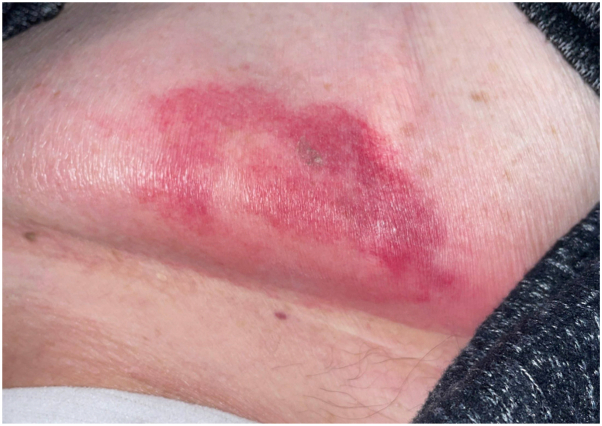
Teclistamab injection site reaction presenting as an erythematous, annular plaque located on the right abdomen.

On evaluation, the patient was afebrile with stable vital signs. Dermatologic examination revealed a single, indurated, erythematous, and purpuric annular plaque on the right abdominal pannus (Fig 1). The lesion was tender to palpation. The remainder of physical examination was unremarkable with no further mucocutaneous lesions identified. Laboratory studies demonstrated normal white count, anemia (Hgb 7.5 g/dL), decreased platelets (406,000/uL), and differential demonstrating an absolute neutrophil count of 4300/uL and absolute lymphocyte count of 200/uL.

**Fig 2:**
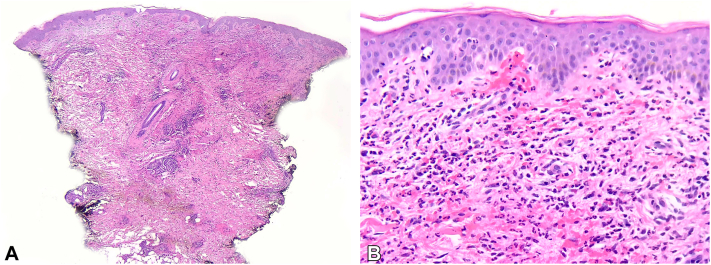
Histopathology of punch biopsy demonstrating infiltrate of neutrophils and lymphocytes extending into deep reticular dermis and superficial subcutis with magnifications of (**A**) 25× and (**B**) 400× (hematoxylin-eosin stain).

Punch biopsy of the plaque demonstrated a moderately dense infiltrate of neutrophils and lymphocytes extending into the deep reticular dermis and superficial subcutis. No necrosis was visualized and the epidermis was unremarkable (Fig 2). Tissue culture was negative with no organisms seen on gram stain, acid-fast bacilli stain and culture, periodic acid–Schiff stain, Grocott stain, and anaerobic cultures. There was no evidence of involvement by myeloma.

The location of a single lesion at the site of the teclistamab injection, the presence of a neutrophilic infiltrate, and the negative infectious workup favored a diagnosis of injection site reaction.

The patient continued step-up dosing of teclistamab and received her second injection on day 4 of admission, avoiding the site of her lesion. No further dermatologic findings were noted with the second injection; however, she developed fevers with workup revealing grade I CRS. Four days following initial dermatology evaluation, the patient was re-examined and found to have significant resolution of her lesion. The patient was discharged after completion of teclistamab step-up dosing with no further complications.

## Discussion

Initial studies have demonstrated promising results for BiTEs, including teclistamab, leading to several antibodies receiving accelerated Food and Drug Administration approval. However, data on the dermatologic effect of BiTEs remain limited.

Elranatamab is a BiTE sharing the same molecular targets as teclistamab (CD3/BCMA). Similar to teclistamab’s dermatologic safety profile, a phase II study of elranatamab reported injection site reactions in 26.8% of patients and no additional studies reporting other cutaneous events.^8^ In contrast, blinatumomab (CD19/CD3; B-cell acute lymphoblastic leukemia), a non-BCMA bispecific T-cell engager, was associated with a variety of cutaneous reactions including eczema, psoriasis, seborrheic dermatitis, acne, folliculitis, dermatitis, and panniculitis.^9^

Given the incidence of DAEs for bispecific T-cell engager therapies across different targets, the reaction in our patient may be attributable to T-cell activation. Parisi et al hypothesized that the high incidence of CRS among BiTEs suggests that T-cell activation may cause a cytokine-mediated dermatologic response.^9^ Additional studies comparing dermatologic events in patients with and without CRS may provide further insight into this potential mechanism.

Shimoda-Komatsu’s review of injection site reactions following azacitidine treatment, a nonbiologic used for multiple myeloma, demonstrated similarities with our report. Azacitidine reactions presented as erythematous patches with histopathology ranging from a neutrophil-predominant to lymphocytic infiltrates.^10^ The similarities in presentation between azacitidine and teclistamab injection site reactions may further support that injection site reactions arise from stimulation of an endogenous pathway, independent of a drug’s particular mechanism. Of note, our patient developed an injection site reaction only after her first dose of teclistamab. In Shimoda-Komatsu’s review, there were several reports without a recurrence after subsequent injections.^10^ Future studies correlating recurrence with histopathology may clarify if there exist true subcategories of injection site reactions or if such reactions present heterogeneously.

Fever and the presence of neutrophils on pathology, as seen in our patient, warrant a consideration of infection and Sweet syndrome. In our patient, the presentation of a single annular plaque with no further skin changes and negative infectious workup was most consistent with an injection site reaction. Overall, the high rate of infections, cytopenias, and CRS among patients receiving BiTE therapy all underscore the importance of a broad differential with thorough evaluation and workup.

As BiTEs are increasingly developed and utilized in the treatment of malignancies, this case report illustrates that further reporting and investigation are needed to develop an understanding of the dermatologic risks of T-cell engagers. Further, given the rates of injection site reactions reported in initial studies, dermatologists should be aware of this potential reaction.

## Conflicts of interest

None disclosed.
